# An environmental health vocabulary and its semi-automated curation workflow

**DOI:** 10.1080/2833373X.2025.2485111

**Published:** 2025-04-22

**Authors:** Michelle Angrish, Scott Burns, Joshua Cleland, Caroline Foster, Samuel Kovach, Kristan Markey, Brittany Schulz, Andy Shapiro, Michele Taylor, George Woodall, Sean Watford

**Affiliations:** a Center for Public Health and Environmental Assessment, Chemical and Pollutant Assessment Division, US Environmental Protection Agency, Durham, NC, USA; b Center for Public Health and Environmental Assessment, Health and Environmental Effects Assessment Division, US Environmental Protection Agency, Durham, NC, USA; c ICF International, Fairfax, VA, USA; d Oak Ridge Associated Universities, Environmental Protection Agency National Student Services Contract, Oak Ridge, TN, USA

**Keywords:** Ontology, controlled vocabulary, health information, systematic review, risk assessment, data curation, artificial intelligence, interoperability/standards

## Abstract

The environmental health vocabulary (EHV) represents manually curated terminologies developed by the US Environmental Protection Agency’s (EPA) Chemical Pollutant Assessment Division (CPAD) for standardizing reporting of health effect information. Recognizing that manual data curation is a resource bottleneck, a semi-automated curation workflow was realized. The objectives of this work are to describe the manual creation of the EHV and improve the efficiency of manual data curation by implementing a new semi-automated curation workflow that minimizes manual review using a sequence of computational text analysis and quality assurance/quality control (QA/QC) steps with a high level of accuracy. To facilitate semi-automated curation a sequence of computational text analysis and manual steps were developed. Described are (1) a series of computational text processing steps to normalize and match extracted terms to the EHV, (2) a QA step of the computationally identified matches; (3) a manual review of unmatched terms; and (4) curation of the EHV that includes completion of missing hierarchical data and related metadata. The EHV was manually created to promote data aggregation, integration, accessibility and transparent data exchange across EPA partners by normalizing the data extracted into the EPA Health Assessment Workplace Collaborative (HAWC). The workflow described here removes the manual curation bottleneck by transforming data curation into a streamlined semi-automated process powered by computational text processing steps. This semi-automated curation method offers several advantages to the environmental health community including (but not limited to) efficiency by automating repeating data management tasks, scalability to a large volume of terms and terminology resources, and better integration with other data sets and artificial intelligence (AI) and machine learning (ML) models.

## Introduction

The U.S. Environmental Protection Agency’s (EPA) Chemical Pollutant Assessment Division (CPAD) utilizes systemic search, selection, and coding strategies to produce searchable databases of study data. These databases include data and descriptive metadata that helps the reader evaluate the reliability and quality of the evidence used in human health chemical assessment. The U.S. EPA has been summarizing these data using data extraction templates, such as those implemented in systematic evidence maps (SEMs) (Thayer et al. 2022). Data extraction templates promote consistency across the extracted study data and foster data accessibility and exchange across the environmental health community. SEMs developed from these templates expedite the assessment process, can be defined as “A comprehensive summary of the characteristics and availability of evidence as it relates to broader themes of policy or decision-making relevance” (Wolffe et al. 2019) without presenting conclusions on the science supporting human health and environmental assessments. Increased utilization of template formats streamlines data exchange and promote efficiencies and cost savings for all partners invested in data gathering, problem formulation, and evidence surveillance. Yet, data extraction and reporting are resource intensive due to the lack of structure and semantic variability in the reported data.

Data extraction is implemented in the U.S. EPA’s version of Health Assessment Workplace Collaborative (HAWC) (https://hawc.epa.gov/vocab/ehv/) as described in the *ORD Staff Handbook for Developing IRIS Assessments* (U.S. EPA. (U.S. Environmental Protection Agency) 2022). Part of the data extraction strategy includes application of agreed (meta)data standards as proactive solutions to normalize and standardize the complex terminologies used to describe the environmental health science domain. For example, there are many textual descriptions data extractors could use to report the study details and findings for a particular finding or health effect (e.g. alanine transferase-Serum Glutamate-Pyruvate Transaminase-Alanine Aminotransferase-Serum Glutamic-Pyruvic Transaminase-Alanine Transaminase-serum glutamate pyruvate transaminase). While these are related, they do not match, leading to inconsistencies that, if not standardized, complicate aggregation and integration of the findings linked within and across chemical assessments and other human and environmental health data.

**Figure 1.**
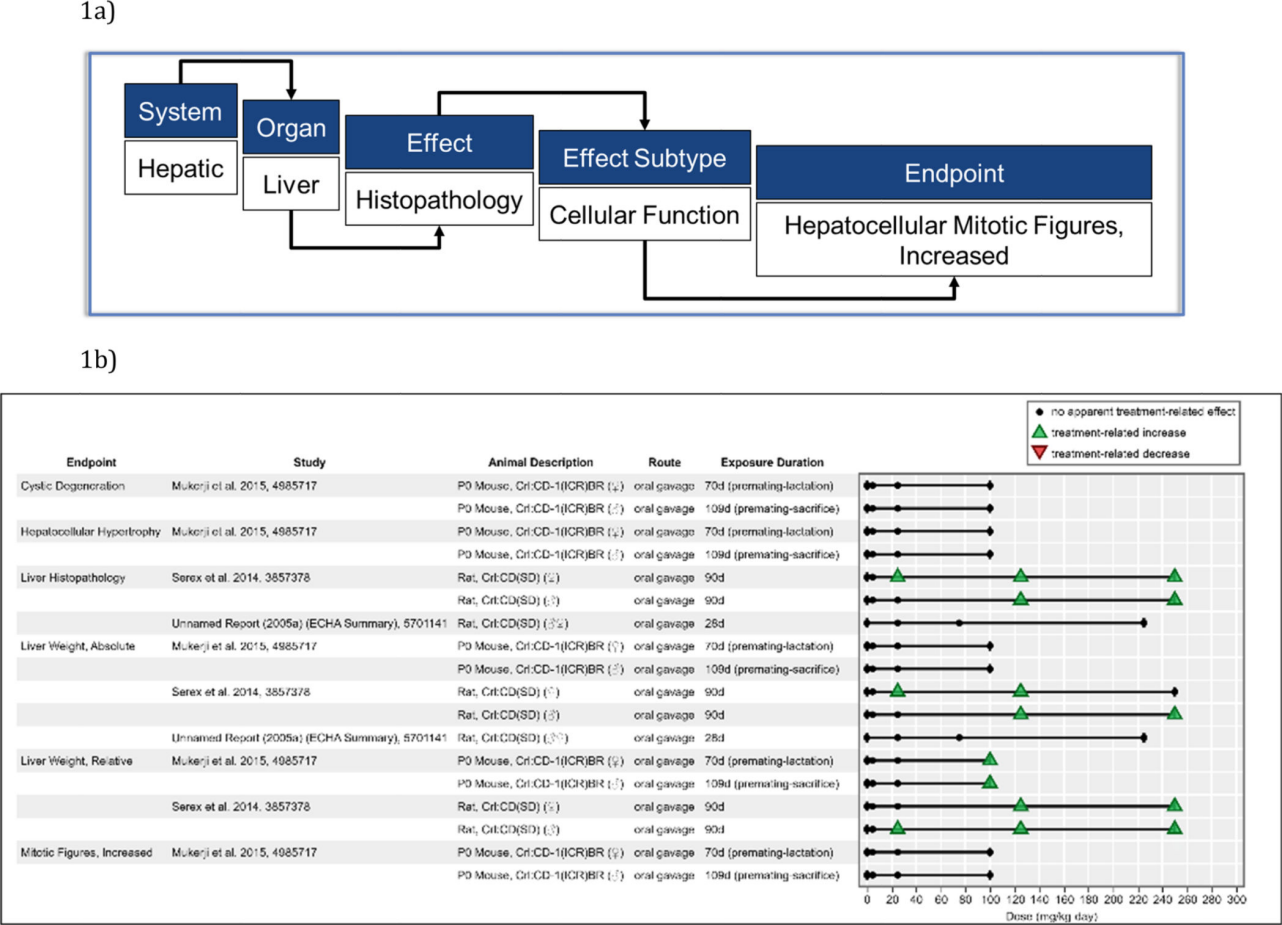
A) The environmental Health Vocabulary (EHV) is a five-level hierarchy to describe health effects within assessments. Currently the EHV describes animal health effects. The only implementation of the EHV is within HAWC and is available at https://hawc.epa.gov/vocab/ehv/. B) The EHV enables a visual summary of endpoints aggregated by health system (hepatic) and organ (liver).

Therefore, the Environmental Health Vocabulary (EHV) was created by EPA CPAD in 2022 to both normalize the reported terminology and standardize endpoint terms extracted into HAWC. Standardization of the “Endpoint” field (meta)data is a significant semantic challenge because the number of available endpoints is vast and, as described above, there are usually multiple ways to describe the term. The EHV is a standardized and controlled vocabulary for reporting animal health effects of environmental chemical exposure. A controlled vocabulary is an organized collection of words and phrases that typically includes preferred terms that are non-redundant, unambiguous, can be indexed, are machine readable and can be used to search a content management system through query (see https://www.niehs.nih.gov/research/programs/ehlc/resources for additional information and resources). The EHV is designed to have a five-tier hierarchical order such that: system->organ->effect->effect sub-type->endpoint/outcome (Figure 1A). The hierarchy supports aggregation of endpoints into visual representations that can be categorized at the system, organ, effect, or effect subtype level depending on assessment need. The EHV can be used as a reference for reporting health effect information primarily from animal bioassay studies and is currently implemented in HAWC. The EHV is user enabled and accessible in the HAWC data entry screen where EHV terms are available for selection by name or unique ID.

The EHV was developed through a manual curation effort of terms and phrases extracted from the peer-reviewed animal toxicology literature (identified during assessment developed based on Population, Exposure, Comparator, and Outcome criteria, PECO) into HAWC as part of the chemical assessment workflow (see Chapter 5). Extraction and Display of results from epidemiological and toxicological studies, U.S. EPA (U.S. Environmental Protection Agency 2022). In the current EHV, the curation process consisted of 100% manual review of the terms extracted into HAWC by PhD level experts with assessment development experience. The manual process involved download of HAWC extracted terms, manual review supplemented by consultation with domain experts as needed, look-up from terminology resources such as Clinical Data Interchange Standard for Exchange of Nonclinical Data (CDISC SEND 2023) Controlled Terminology, Unified Medical Language Syntax (National Library of Medicine 2023), and (Makris et al. 2009) for developmental and reproductive outcomes and comparison to prior health endpoint mappings from the U.S. EPA’s Integrated Science Assessments. As described above, the exact organization of the EHV and terms within the EHV were developed to improve the efficiency of data aggregation, integration, and filtering for visualization of the findings that support animal evidence synthesis and visualization in human and environmental health assessments. For example, the EHV enables data aggregation and visualization across multiple studies by health system, organ, and endpoint (Figure 1B). The EHV is optional, allowing for free text data entry when a newly extracted term is not available in the EHV. Approximately half of all public EPA HAWC projects are using the EHV, and within EHV enabled projects, approximately 37,000 endpoints were extracted (last updated December 2024). Of those, approximately 70% of all endpoints used the EHV directly, with 30% using custom user defined terms.

Manual curation is a time-consuming data extraction process requiring expert technical insight. Therefore, the objective of the EHV Curation Workflow described herein is to minimize the requirement of manual review and analysis of extracted terms by applying a sequence of computational text analysis steps with a high level of accuracy. Further, several steps in the curation workflow (term matching and look-up) include text mining steps can be scaled-up and semi-automated using computational approaches to streamline the data extraction process.

## Methodology

### Workflow overview

The following terms associated with the workflow described here are defined in Box 1: Term, curate, standardize (or normalize), match (matching), and cleanup. Terms without a confirmed match are candidates for further manual review and possible addition to the EHV.

Because terms in the EHV are organized within a five-level hierarchy (i.e., organ system, organ, effect, sub-effect, and endpoint), curation involves normalizing the endpoint terms and then the associated terms falling into each of the other four hierarchical levels. Curation also involves completing missing definitions and other metadata using unified medical language system (UMLS), clinical data interchange standards consortium-standard exchange for nonclinical data (CDISC-SEND), medical subject headings (MeSH), logical observation identifiers names and codes (LOINC), Uber-anatomy ontology (UBERON), etc.

The EHV curation workflow is intended to semi-automate curation steps while including a human in the loop. Terms are initially processed and grouped based on whether the term is a new or existing term. The specific details and subsequent steps of the workflow are described below.

At high level, the workflow compares terms extracted into HAWC to the existing EHV and sorts them through the workflow depending on if they match or do not match the EHV. Terms that do not match are candidates for additional curation and possible addition to the EHV. Terms extracted into HAWC are identified as matches if the exact term is already in the EHV or if the EHV has a synonym, another term, or phrase that represents the same concept. Any synonymous matching terms and phrases can be used to refine the efficiency of the workflow when applied to future sets of extracted terms. These terms are compiled into a “cumulative equivalents dataset”. Matching terms enter QA/QC. Terms that do not match the EHV after the computational text processing step proceed to manual and manual review before QA/QC.

**Figure 2.**
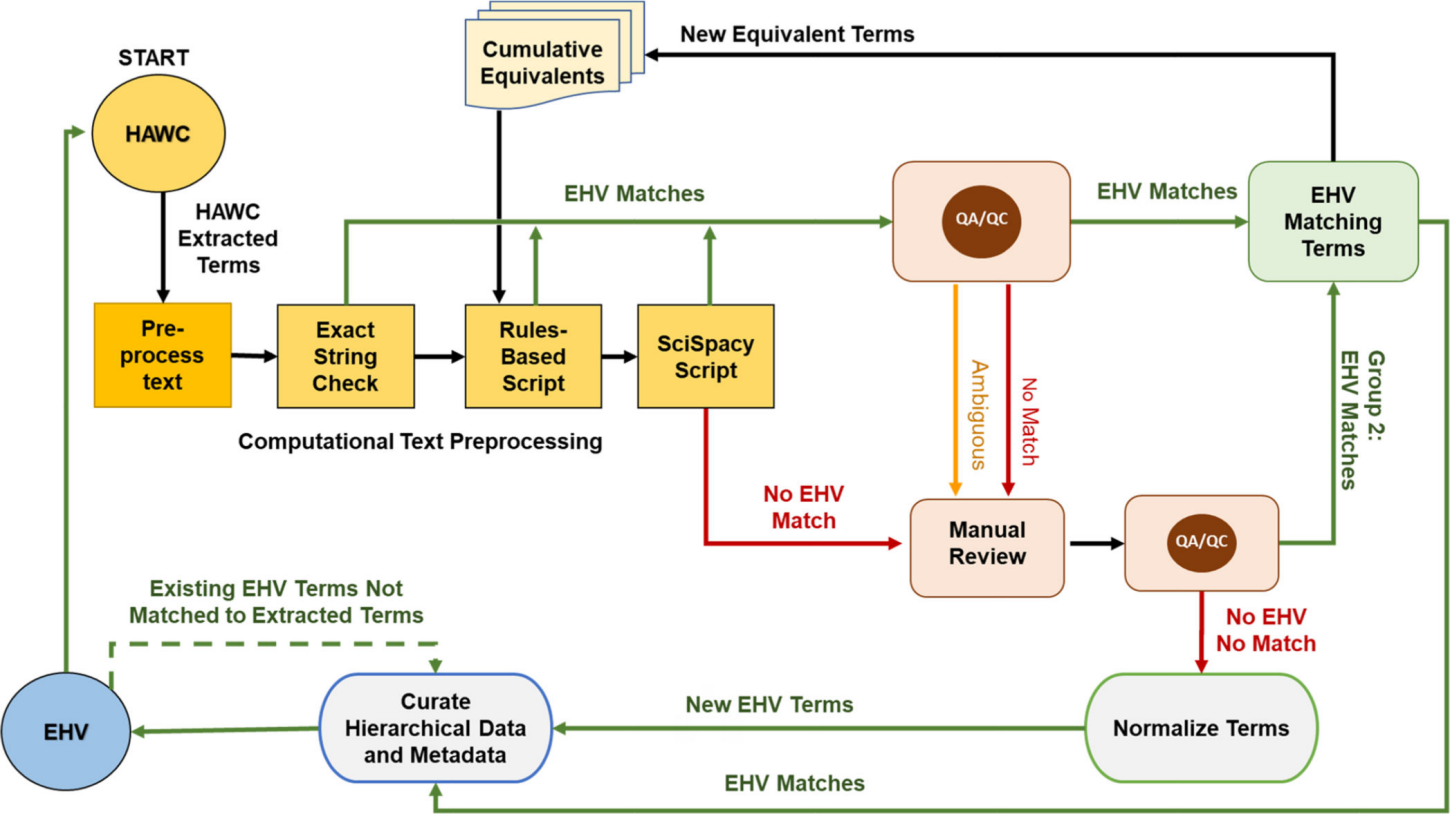
Environmental health vocabulary (EHV) curation workflow. The curation workflow is presented in stages that include computational text processing (preprocess text, exact string match, rules-based script, and SciSpacy), QA/QC of computational matches, manual review and matching followed by QA/QC, and curation of the EHV data.

Figure 2 provides an overview of the EHV Curation Workflow for terms extracted into HAWC. The workflow begins on the left with a set of HAWC extracted terms.

The workflow includes four distinct stages:

1. Computational text processing to clean-up extracted terms (e.g., remove punctuation and extraneous characters) and identify matches to existing EHV terms.

2. QA/QC of the computationally identified matches, including the use of hierarchical data to aid in binning terms and manual QA/QC review.

3. Manual review and matching of unmatched terms to identify additional matches that were not identified by computational processing.

4. Curation of EHV data, including the normalization of terms and completion of hierarchical data and metadata.

Each of these stages are discussed in more detail in the sections below.

### Computational processing

The computational processing stage within the workflow is shown in Figure 1 by the yellow rectangular boxes. As the name implies, computational processing is carried out with a series of Python scripts available in the GitHub Repo. To run these specific scripts without modification, the input data must be formatted consistently.

Four steps are carried out within this stage:

- Text pre-processing
- Exact string matching
- Rules based matching; and
- SciSpacy (Neumann et al. 2019), a Python package containing spaCy (https://spacy.io/ models for processing biomedical, scientific or clinical text entity matching).

All steps are applied to terms extracted into HAWC and input into a consistent file format. (Note that MS Excel is the file type used in this workflow so any change in file type will require update to the scripts). Each of the scripts uses the pandas (V2.2.3) Python library to read the Excel information as a data frame. Several functions are written in each of the scripts that can be applied to a column using panda’s “apply” method. Other scripts may be written as loops that utilize panda’s itterows method of iterating. To rerun the scripts, a user supplies the data files (the terms extracted into HAWC and the EHV inputs as MS Excel spreadsheets) and updates the scripts to the correct file paths to the data file(s). (Note that example data files are provided in the GitHub repo.) The column names need to match the original column names or be updated throughout the script. When data are transformed by the scripts, the resulting text is copied to a new column so that the original and transformed data can be viewed side-by-side for documentation and QA/QC.

### Text pre-processing

The goal of text pre-processing is to enhance the machine readability of the extracted terms for the subsequent steps. That is, the terms are “cleaned up” to improve the efficiency with which they can be matched to the EHV. The pre-processing code applies distinct transformations to achieve this goal:

- Numeric codes at the end of a string, often starting with “XXX” or numbers alone, are removed.
- Punctuation is separated from text characters by adding spaces before and after each punctuation character. Dashes or hyphens within the text are replaced with spaces.
- Leading and trailing spaces (i.e., spaces preceding and following the text, respectively) are removed and extraneous spaces within the text are replaced with a single space.
- All text characters are converted to lowercase to avoid case-related discrepancies during matching.
- Parentheses and brackets are removed, along with their contents, to minimize extraneous information that may interfere with matching.
- Words are lemmatized, i.e., reduced to their base form.

The resulting cleaned text is stored in the “1_cleaned” column of the Excel dataset. Specific examples for of text preprocessing of the terms “Alanine Aminotransferase” “Excitation”, and “Live Fetuses/Litter” are given in Table 1.

### Exact string matching

After pre-processing, the exact string-check step searches for an exact match in the EHV for each HAWC extracted term. This step uses the endpoint terms only; hierarchical data, definitions or other contextual information is not used. Results are stored in the “EHV_ID” and “Endpoint” columns. When a match is identified, the EHV ID and Endpoint name are added to the “EHV_ID” and “Endpoint” columns, respectively. The term is added into the original Excel file as shown in Table 2 (note that if there is no match the entry will be blank).

### Rules-based matching

The rules-based matching step uses text analysis algorithms to identify matching endpoint terms when the HAWC extracted terms do not match the EHV term. The algorithms find matches by evaluating specific words or phrases and synonyms of those words and phrases. Extracted terms are matched to synonymous terms identified with the Natural Language Toolkit (NLTK, V3.9.1) Python library (Bird, Loper, and Klein 2009). Via a separate script, synonyms for individual words and phrases are obtained using functions in NLTK through its WordNet module, which is based on a database of English words and conceptual relationships between words such as antonyms and synonyms. Synonyms identified by NLTK resources are exported as a Python dictionary for use in rules-based matching.

The script then searches for matches between words as well as between words and their synonyms. When a match is found (either exact match or through a synonym), the EHV ID and Endpoint name are added to the columns “EHV_ID” and “Endpoint”, respectively. Table 3 shows the results of the rules-based matching of HAWC extracted terms. If no match is found the cell is blank.

### SciSpacy entity matching

The final step of the Computational Processing stage of the workflow leverages the capabilities of SciSpacy, an open-source software library for advanced natural language processing. SciSpacy is specifically adapted for the analysis of scientific and biomedical text sources (Neumann et al. 2019). Within the workflow, SciSpacy is used to link the terms in each dataset (i.e., terms extracted into HAWC and EHV terms) to equivalent scientific entities (i.e., concepts). The objective of this step is to map the identified entities within the text to their corresponding Concept Unique Identifiers (CUIs) from the Unified Medical Language System (UMLS) Metathesaurus (Bodenreider 2004). The UMLS Metathesaurus is a comprehensive source of biomedical and health-related concepts, terms, and codes.

The “SciSpacy” library employs sophisticated natural language processing techniques to recognize entities within the text that are relevant to the concepts in the biomedical domain. These entities could include medical terms, diseases, drugs, or anatomical structures. Once identified, the library links these entities to specific Concept Unique Identifiers (CUIs) within the UMLS Metathesaurus, providing a standardized way to represent and reference biomedical concepts. Table 4 shows the results of the SciSpacy of HAWC extracted terms. If no match is found the cell is blank. The full results of computational data processing are available in the GitHub repo.

### QA/QC of computational matches

Outputs from the computational processing stage include terms that matched the EHV and terms that do not match. Terms that match the EHV proceed into the QA/QC stage of the EHV Curation Workflow. The purpose of this stage is to provide quality assurance (QA/QC) for the computational matching of the extracted terms to EHV terms. This QA/QC only pertains to terms that have been identified as matches by the computational processing steps. Terms that were not identified computationally as matches proceed to full manual review and are evaluated in a separate sequence of steps that is described under the “Manual Review and Matching” section below.

**Figure 3.**
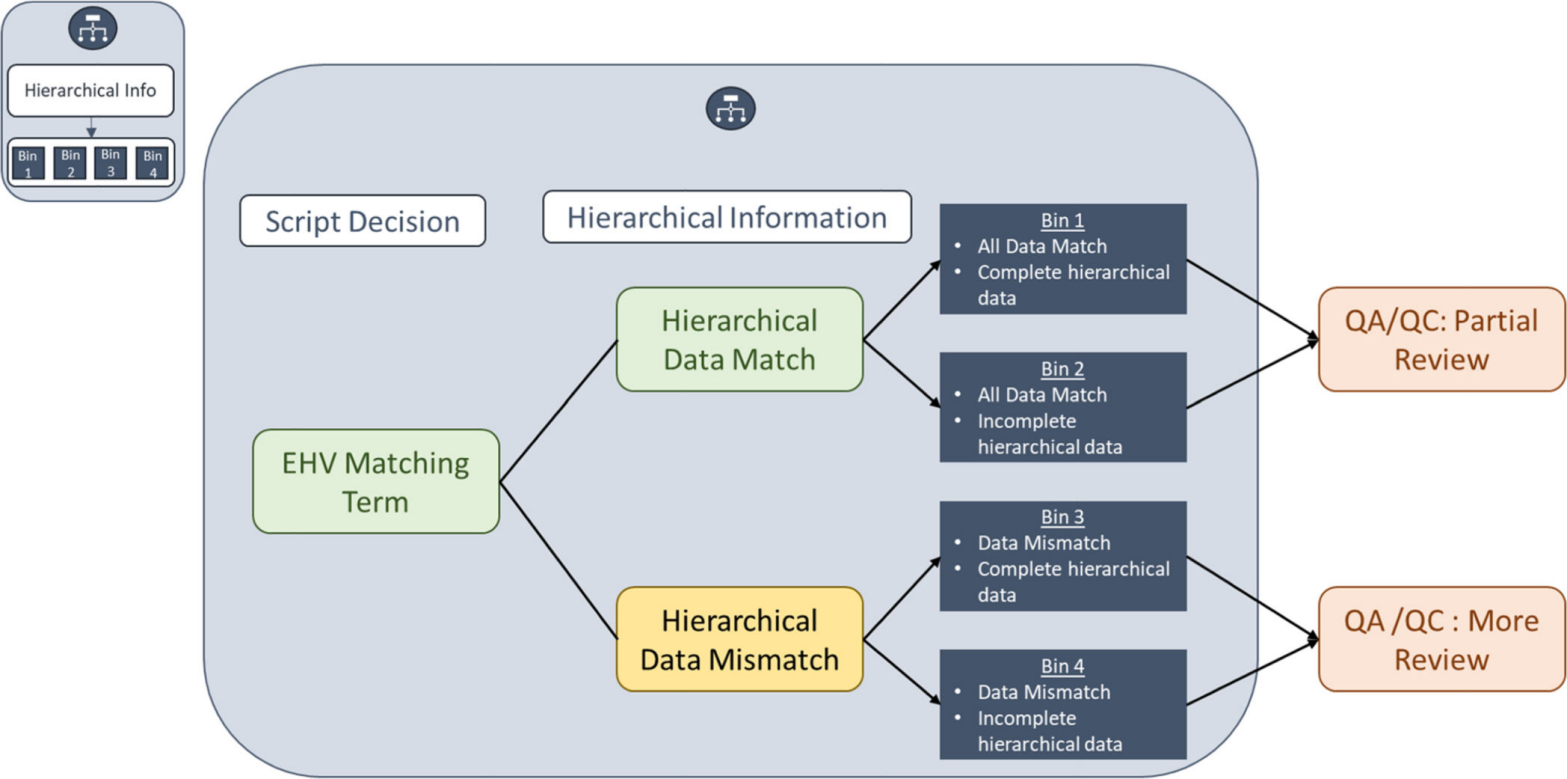
QA/QC of matching terms. Hierarchical data enable to terms to be sorted into four bins based on the completeness of the hierarchical data and the data match/mismatch. Bins 1 and 2 receive a partial review whereas bins 3 and 4 receive a more complete review.

Because hundreds or thousands of extracted terms may be processed through the workflow at once, the QA/QC review of individual terms can be time consuming. Therefore, to minimize the resources required to perform QA/QC, the hierarchical data associated with each of the terms are used to further evaluate the likelihood of correct matching. The inclusion of hierarchical data enables the terms to be sorted into four groups (i.e., “bins”, Table 5 and Figure 3). Bin 1 represents instances where hierarchical data are complete and all data matches. Bin 2 represents instances where all data matched, but hierarchical data are incomplete. With this approach, partial QA/QC can be performed for bins 1 and 2 (all data match) whereas full review is required for bins 3 and 4 (data mismatch) (Table 5). This approach minimizes the overall level of effort required for expert-level resources required for manual QA/QC.

Hierarchical data are compared and binned electronically based on two factors: (1) whether complete hierarchical data are available for the potential term matches and (2) whether the available hierarchical data match. Hierarchical data are complete only if data are available for all five levels (i.e., system, organ, effect, sub-effect, and endpoint) in both the EHV and HAWC extracted terms datasets.

The EHV and HAWC extracted term datasets are compared electronically and classified as true (matching) or false (non-matching). The true/false classifications are written into the Excel dataset as a new column named “Stg# Match as true (1) or false (0)” to aid reviewers in sorting and prioritizing records for QA/QC review. During development of this workflow, it was observed that a majority of the terms fell into bins 1 (all data match and hierarchical data were complete) and 3 (data mismatch, but hierarchical data were complete). It was also observed that the data mismatches were mostly due to transposition of hierarchical data fields. For example, effects and sub-effects were often swapped, causing a mismatch. Therefore, the current matching approach ignores the order of the hierarchical levels when evaluating matches. For these bin 3 instances, the assumption is that the hierarchical levels in the EHV are correct. However, QA/QC reviewers should record recommendations to reorder the EHV hierarchical data when appropriate.

For the most confidently matched terms in Bins 1 and 2, a single reviewer will review a random sample representing 10% of the matched terms. Discrepancies identified by the first reviewer will be reviewed and resolved by a second reviewer. If discrepancies are found in more than 25% of the high confidence terms sampled, the review steps will be repeated for a second, non-overlapping 10% sample of the terms. This approach will be repeated until fewer than 5% of the terms are identified as discrepancies between reviewers. Based on the performance of the computational text in full scale implementation of the workflow, the proposed QA/QC schema may be adjusted to appropriately balance quality control and the level of effort required for manual review. A similar approach will be used for the less confidently matched terms in bins 3 and 4 except the reviewer will review 20% of the terms computationally matched to the EHV. If discrepancies arise during QA/QC that cannot be resolved by the primary and secondary reviewers, those terms will be classified as ambiguous and will proceed to expert manual review.

### Manual review and matching

The manual review and matching stage in the EHV curation workflow (Figure 1) apply to terms where computational processing did not result in any matches to existing EHV terms. Whereas QA/QC review described in the previous section compares paired sets of terms and hierarchical data to confirm matches, the purpose of manual review in this stage is to identify matches for those terms that computational methods failed to identify. To do this, expert reviewers compare 100% of the unmatched extracted terms to the EHV. The reviewers decide whether each extracted term matches an existing EHV term, has no match, or has inadequate data to support a decision (i.e., ambiguous). Extracted terms that the reviewers determine do not match any existing EHV terms will be considered as candidates for addition to the EHV.

When performing manual review and matching, expert reviewers will compare terms as well as the hierarchical data associated with the EHV and extracted terms, as available. Comparing the hierarchical data provides the expert reviewers with additional context to assess whether the endpoint terms represent the same concept. For example, nearly equivalent terms will be considered nonmatching if they are associated with different organs or organ systems in the EHV and extracted term datasets. In addition, expert reviewers can filter the EHV data by hierarchical data fields to search for matches more efficiently. If an extracted term is associated with a particular hierarchical category (e.g., cardiovascular system), the reviewer can filter the EHV terms so that only cardiovascular endpoints are shown for comparison. To increase the efficiency of this step, an electronic reviewer form or other tool may be developed or adopted in the future to facilitate filtering and side-by-side data comparisons.

This manual review and matching stage will require expert knowledge of toxicological studies, including study and exposure design, study methodology, human and animal physiology, and toxicological health endpoints. Therefore, manual review will be based on domain expert judgement due to incomplete hierarchical data, text and conceptual ambiguities, and other data limitations. The basis for expert judgments will be documented by reviewers as explanatory comments when appropriate to make transparent the rationale for reviewer decisions in a suitable ontology management software.

For quality control, it may be advisable to require independent review of the matching decisions by a second expert reviewer. The QA/QC approach, scope, and level of review should be decided following a preliminary (e.g., spot-check) review of the initial matching and QA/QC results, including the reviewer comments, the number of terms in the data set, and other factors. For example, depending on the results of the preliminary review, full QA/QC of the expert review could require a second, independent review of all terms, secondary review of a sample of terms, or secondary review focused on matched terms, non-matched terms, or terms considered inconclusive by the primary reviewer. Discrepancies between the primary and secondary reviewers should be resolved by a third reviewer.

The EHV Curation Workflow sorts HAWC extracted terms into two groups, including terms found to have matches already in the EHV and terms determined to be absent from the EHV. The process for curating these term groups to maintain the EHV is discussed below.

### Terms with EHV matches

A primary objective of the EHV Curation Workflow is to identify health endpoints that can be added to the EHV. As shown in Figure 2, terms that are matched to EHV terms follow the pipeline to curate hierarchical data and metadata. Terms that are already represented in the EHV are excluded from further consideration, although these terms and associated hierarchical data are useful to consider for refining the EHV. In the EHV curation steps, the hierarchical and other data associated with the matching extracted terms can be used to complete EHV information for the existing terms. A detailed sub workflow for this step has not yet been developed, but this sub workflow is expected to reference existing resources such as the UMLS for missing information. Missing information may include endpoint terms that lack complete hierarchical data, definitions, and identifiers such as UMLS concept unique identifiers (CUI) and/or unique identifiers of semantic types (TUI), when available. As indicated by the dashed line in Figure 2, the sub workflow would apply for curating hierarchical data and metadata to existing EHV terms that were not matched to any of the extracted terms that entered the workflow.

The continued curation of EHV matching terms could be used to refine the efficiency of the computational processing stage of the workflow. Specifically, new equivalent terms may be identified from the EHV matching terms, which can be added to the cumulative equivalents dataset library. With each application of the EHV Curation Workflow, extracted terms determined to equate to existing EHV endpoints, along with their associated data and EHV counterparts, are added to this cumulative equivalents dataset. In subsequent applications of the workflow, the rules-based script will compare extracted terms entering the workflow to the cumulative equivalents dataset to find EHV matches that were identified by previous application of the workflow to sets of extracted terms. This approach may incrementally increase the efficiency and confidence of semi-automated, rules-based matching when the workflow is applied to new sets of extracted terms. Rules-based matching using the cumulative equivalents dataset has not yet been developed but could be added to the existing workflow as a potential next step.

### Terms without EHV matches

The EHV curation workflow employs a series of computational and manual steps to match extracted terms to EHV endpoints. Terms that are unmatched after the completion of the workflow may be potential new endpoints for addition to the EHV. As part of the expert review steps reviewers will classify unmatched terms as either eligible health endpoints or ambiguous terms (e.g., not health endpoints). Eligible endpoints recommended by the reviewers will undergo additional review to confirm induction as new EHV endpoints.

As shown in Figure 2, the terms without EHV matches progress toward addition to the EHV via the normalize terms step. Normalizing a new EHV term means selecting the precise word or phrase that will be used to consistently represent the new health endpoint concept. Normalization may be needed if there are various forms (i.e., plural or singular) of words or phrases that are commonly used to represent the same concept (e.g., white blood cells, WBC, monocytes or alanine aminotransferase, ALT, serum glutamic, pyruvic transaminase, SGPT). To promote interoperability among tools and datasets, the UMLS and/or other relevant ontologies will be used to identify normalized terms and phrases, as available. If normalized terms are not found in other relevant ontologies, the most appropriate form of the term will be selected for use in the EHV.

### Challenges and limitations

Currently implementation of the workflow is limited because some EHV endpoints are missing metadata and/or definitions. For example, SciSpacy uses metadata and definitions to link endpoints to biomedical ontologies and other knowledgebases (including UMLS). If these metadata and definitions are missing, SciSpacy may fail to correctly identify correct terms. Further, missing metadata and definitions provide context, that when missing, can lead to inaccuracies in linking relationships between terms and in identifying correct concepts from probed knowledgebases. Through iterative curation of the EHV, it is expected that these data gaps will lessen, increasing performance of the computational text processing steps and increase efficiency by reducing the overall time needed for manual curation. Therefore, a critical next step will include data gap filling (including hierarchical metadata and definitions) of the current EHV.

The need for domain expertise is also a possible challenge to the workflow. Non-experts may lack knowledge on the current standards and conventions in environmental health science and struggle to accurately curate terms possibly leading to inconsistencies, data gaps, ambiguity, missing hierarchical data, and or missing definitions. These inaccuracies could feedback on computational processing steps, impeding performance or introducing inaccuracies that decrease trustworthiness and interoperability with other terminology resources. Therefore, agreed upon data standards for vocabularies and ontologies within a complex domain are proactive solutions that can be adopted by a community working together to ensure consistency, quality, accessibility, and exchange of data. The true challenge will continue to be the continued curation of these metadata and maintenance of a pool of domain experts as data sets and ontologies are developed. A decentralized model for data curation within a trusted community of domain experts is needed to bridge knowledge gaps and may be achieved through training, community building, tooling, and incentives.

### Next steps

Implementation of the EHV Curation Workflow is currently undergoing additional development and testing to include automation tools using an ontology management software application. For this use case the Graphite Taxonomy and Ontology Management system was selected as the software application to manage both the source vocabulary as well as the curation. The ontology management software is specific for ontologies and allows modeling from multiple terminology resources, capturing provenance during curation while allowing terminology changes back to other software (such as HAWC) after curation. Through implementation of the curation workflow, detailed sub workflows will be developed for curation and standardization of hierarchical metadata for EHV terms. The metadata is needed to improve the performance of some of the computational scripts (e.g. SciSpacy) that are currently limited by lack of metadata such as term definitions that will additionally expand the computational processing stage to match extracted terms using the cumulative equivalents dataset. Anticipated improvements will also be realized as the ontology software should provide a user-friendly interface for QA/QC that is web accessible, possibly allowing crowdsourcing and asynchronous collaboration. Since the EHV is the language standard for reporting animal health effects information in human health assessments conducted by EPA CPAD and is made publicly available as a download as well as via the publicly available HAWC application, it is envisaged that the EHV can help to standardize the way findings are reported across the environmental health community. We anticipate engaging with the broader environmental health science community to ensure not only standardization of the language, but also coverage of biomedical science and other knowledge domains reported in the literature, but not yet covered by the EHV. The EHV is currently machine actionable meaning that it is “AI ready” We anticipate future updates to the curation workflow will incorporate generative AI to take a deeper dive into the more specific topics that describe environmental health science.

## Figures and Tables

**Table 1.** Text preprocessing of HAWC extracted terms.

HAWC extracted term		Text preprocessed 1_cleaned
HAWC_ID	name	

7713	Alanine aminotransferase	Alanine aminotransferase
1570	Excitation	Excitation
152	Live fetuses/litter	live fetuses/litter

**Table 2.** Text preprocessing and exact string matching.

AWC extracted term		Text preprocessed 1_cleaned	Exact string matching	
HAWC_ID	Name		EHV_ID	Endpoint

7713	Alanine Aminotransferase	Alanine aminotransferase	2317	Alanine aminotransferase (ALT)
1570	Excitation	excitation		
152	Live fetuses/litter	live fetuses/litter	1740	Live fetuses/litter

**Table 3.** Text preprocessing, exact string matching, and rules-based matching of HAWC extracted terms.

AWC extracted term		Text preprocessed 1_cleaned	Exact string matching		Rules based matching	
HAWC_ID	Name		EHV_ID	Endpoint	EHV_ID	Endpoint

7713	Alanine Aminotransferase	Alanine aminotransferase	2317	Alanine Aminotransferase (ALT)	2317	Alanine Aminotransferase (ALT)
1570	Excitation	Excitation			3213	Inflammation
152	Live fetuses/litter	Live fetuses/litter	1740	Live fetuses/litter		

**Table 4.** Text preprocessing, exact string matching, rules-based matching, and SciSpacy of HAWC extracted terms.

AWC extracted term		Text preprocessed 1_cleaned	Exact string matching		Rules based matching		SciSpacy	
HAWC_ID	name		EHV_ID	Endpoint	EHV_ID	Endpoint	EHV_ID	Endpoint

7713	Alanine aminotransferase	Alanine aminotransferase	2317	Alanine aminotransferase (ALT) 971	2317	Alanine aminotransferase (ALT) 971	2317	Alanine aminotransferase (ALT)
1570	Excitation	excitation			3213	Inflammation 3360		
152	Live fetuses/litter	Live fetuses/litter	1740	Live fetuses/litter			1740	Live Pups born

**Table 5.** Proposed schema for manual expert QA/QC of computationally matched terms.

Bin	Hierarchical data are complete?	Hierarchical data match?	Matching confidence	QA/QC requirement

Bin 1	Yes	Yes	Higher confidence-Less manual	10% by single reviewer; discrepancies resolved with second
Bin 2	No	Yes	review needed	reviewer; repeat if >5% discrepancies
Bin 3	Yes	No	Lower confidence-More manual	20% by single reviewer; discrepancies resolved with second
Bin 4	No	No	review needed	reviewer; repeat if >5% discrepancies

## Data Availability

The data that support this work are openly available in HAWC Health Assessment Workspace Collaborative (HAWC) (epa.gov) and github repo GitHub—USEPA/ehv: A repository for curation and related activities of the Environmental Health Vocabulary (EHV). The Environmental Health Vocabulary is available at: https://hawc.epa.gov/vocab/ehv/.

## Box 1. Useful Definitions

**Term** – A word, phrase, or variant representing something specific and presented as a single datapoint. For this project, terms are health endpoint names.

**Curate** – To process a dataset of terms by removing extraneous terms or records, normalizing inconsistencies among datapoints within a single field, and adding or correcting required datapoints, identifiers, or metadata.

**Standardize (or normalize)** – To reduce an array of terms that all represent the same thing or concept to a single representative term that is used preferentially.

**Match (matching)** – Computationally or manually determining that a term is equivalent in meaning to a standardized term or that a term can be associated with an established corresponding identifier (ID).

**Cleanup** – Computationally or manually removing extraneous spaces, characters, or punctuation from a collection of terms.
